# Current Knowledge of Individual and Combined Toxicities of Aflatoxin B1 and Fumonisin B1 In Vitro

**DOI:** 10.3390/toxins15110653

**Published:** 2023-11-13

**Authors:** Xiangrong Chen, Mohamed F. Abdallah, Xiangfeng Chen, Andreja Rajkovic

**Affiliations:** 1 Department of Food Technology, Safety and Health, Faculty of Bioscience Engineering, Ghent University, 9000 Ghent, Belgium; mohamed.fathi@ugent.be (M.F.A.); andreja.rajkovic@ugent.be (A.R.); 2 Shandong Analysis and Test Centre, Qilu University of Technology (Shandong Academy of Science), Jinan 250014, China; xiangfchensdas@163.com

**Keywords:** mycotoxins, aflatoxin B1, fumonisin B1, combined toxicity, HepG2 cells, cell apoptosis, mitochondrial toxicity

## Abstract

Mycotoxins are considered the most threating natural contaminants in food. Among these mycotoxins, aflatoxin B1 (AFB1) and fumonisin B1 (FB1) are the most prominent fungal metabolites that represent high food safety risks, due to their widespread co-occurrence in several food commodities, and their profound toxic effects on humans. Considering the ethical and more humane animal research, the 3Rs (replacement, reduction, and refinement) principle has been promoted in the last few years. Therefore, this review aims to summarize the research studies conducted up to date on the toxicological effects that AFB1 and FB1 can induce on human health, through the examination of a selected number of in vitro studies. Although the impact of both toxins, as well as their combination, were investigated in different cell lines, the majority of the work was carried out in hepatic cell lines, especially HepG2, owing to the contaminants’ liver toxicity. In all the reviewed studies, AFB1 and FB1 could invoke, after short-term exposure, cell apoptosis, by inducing several pathways (oxidative stress, the mitochondrial pathway, ER stress, the Fas/FasL signaling pathway, and the TNF-α signal pathway). Among these pathways, mitochondria are the primary target of both toxins. The interaction of AFB1 and FB1, whether additive, synergistic, or antagonistic, depends to great extent on FB1/AFB1 ratio. However, it is generally manifested synergistically, via the induction of oxidative stress and mitochondria dysfunction, through the expression of the Bcl-2 family and p53 proteins. Therefore, AFB1 and FB1 mixture may enhance more in vitro toxic effects, and carry a higher significant risk factor, than the individual presence of each toxin.

## 1. Introduction

Mycotoxin contamination in food represents serious threats toward public health [1]. Mycotoxins are known as toxic secondary metabolites, produced by several toxigenic fungal species, which invade agricultural/farm produce, under certain favorable environmental conditions [2]. Currently, more than 400 mycotoxins (including aflatoxins, citrinin, culmorin, ochratoxins, fumonisins, patulin, zearalenone, diacetoxyscirpenol, sterigmatocystin, nivalenol, T-2, HT-2, deoxynivalenol, enniatins, beauvericin, moniliformin, fusaproliferin, fusaric acid, mycophenolic acid, alternariol, alternariol monomethyl ether, tenuazonic acid, and ergot alkaloid) have been documented from a wide array of toxigenic fungal species, from *Aspergillus*, *Fusarium*, *Penicillium*, and *Claviceps purpurea* genera [3]. Among them, aflatoxin B1 (AFB1) and fumonisin B1 (FB1) are the most prominent compounds linked to a variety of serious human health disorders [4,5].

**Figure 1:**
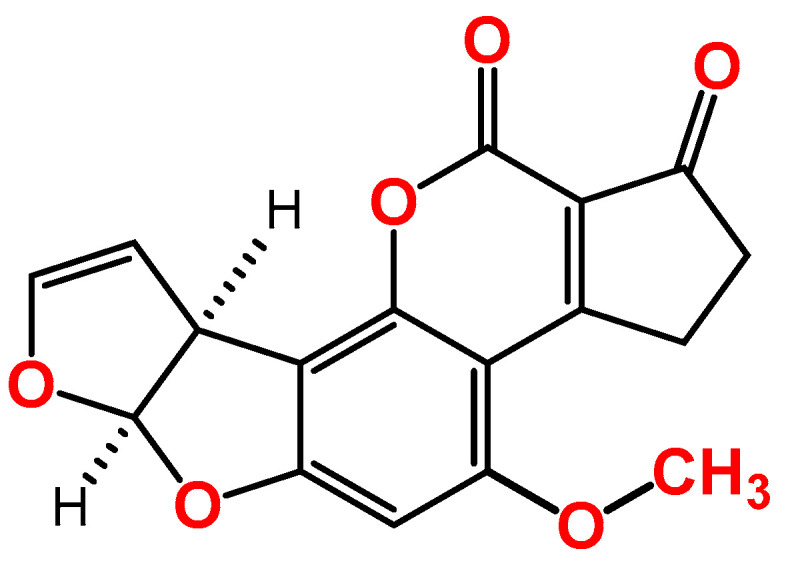
Chemical structure of aflatoxin B1.

AFB1 is a difuranocoumarin derivative (Figure 1), produced mainly by toxigenic *Aspergillus flavus* and *Aspergillus parasiticus* species, and it contaminates different crops, such as nuts, dried fruits, oilseeds, and maize and other cereals. Since the discovery of AFB1 in 1960, after the famous incidence where it killed 100,000 young turkeys in the UK, which was called, at that time, Turkey X disease, several fatal outbreaks have been associated with the consumption of AFB1-contaminated food, as reported in India (the states of Gujrat and Rajasthan in 1974) and in Kenya (Eastern and Central Provinces, in 2040 [6]). The International Agency for Research on Cancer (IARC) classified AFB1 as a carcinogenic agent (group 1 carcinogens), due to its potent hepatocellular carcinoma (HCC) in human [7]. Other toxic effects of AFB1 include immunotoxic, mutagenic, and teratogenic properties in humans [8,9,10]. To protect the public against these effects, several national and international organizations have set regulatory limits for many mycotoxins in different food commodities, according to several factors, such as the toxic effect, contamination rate, and exposure. For instance, the European Union (EU) has set different regulatory limits for AFB1 in ready-to-eat dried figs (6 μg/kg), different types of nuts (5 μg/kg for hazelnuts and Brazil nuts; 8 μg/kg for almonds, pistachios, and apricot kernels; and 2 μg/kg for groundnuts), maize (2 μg/kg), and dried spices (5 μg/kg) [11].

**Figure 2:**
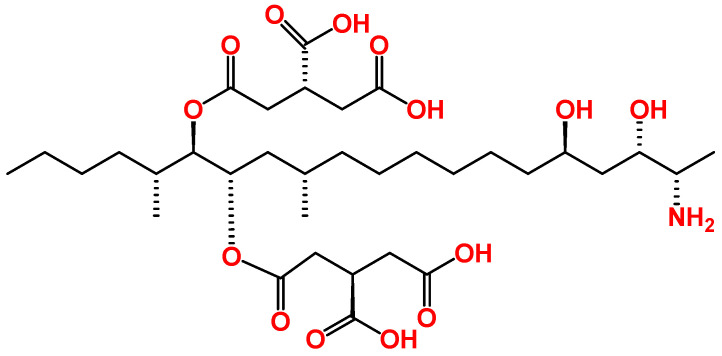
Chemical structure of FB1.

FB1, a sphingosine analogue compound (Figure 2), was the first member of the fumonisin family to be described and characterized, in 1988, after isolation from the *F. moniliforme* MRC 826 fungus. The toxin is mainly produced by *Fusarium verticillioides* and *Fusarium proliferatum* species in cereals, including in corn (maize) and corn-based foods, but also in other cereals, such rice, oat, rye, barley, and wheat [12,13], and several foodborne outbreaks due to the consumption of FB1-contaminated food have been reported over the years in the world [14]. The IARC classified FB1 as a class 2B carcinogen (possible human carcinogen) [15]. It was suggested that FB1 could be associated with the incidence of esophageal cancer in humans in some areas of the world where FB1-contaminated maize is consumed daily, such as South Africa, Iran, and China [16,17]. The toxin poses other toxic effects, such as immunotoxicity, hepatotoxicity, and nephrotoxicity. The EU has set a maximum limit of 2000 μg/kg for the sum of FB1 and FB2 in processed maize for the final consumer [11]. Recently, the European Food Safety Authority (EFSA) has lowered the tolerated daily intake of FB1 to 1 μg/kg bw/day [11,18].

Both AFB1 and FB1 can co-occur in a variety of agricultural commodities, especially maize [19,20]. Therefore, humans are frequently co-exposed to both toxins on a daily basis. This co-exposure is likely to increase in the future when considering climate change as it is expected that the above mentioned mycotoxin-producing fungal species will be more toxigenic and, therefore, produce more AFB1 and FB1 at higher levels than those usually detected in the last decades [21]. In general, the co-exposure to two or more toxins may lead to additive, synergistic, or antagonist toxic effects [22,23,24]. EFSA has already developed some approaches for the exposure assessment of multiple pesticides and other contaminants in humans. Yet, the question regarding what the toxic outcome would be from the co-exposure to AFB1 and FB1, at different doses or scenarios of exposure, still remains unanswered.

The toxicities of AFB1 and FB1 have been studied by many scientists in laboratory animals, as well as in in vitro cell lines and models. However, considering ethical and more humane animal research, the 3Rs (replacement, reduction, and refinement) principle has been implemented by international legislation and regulations. The main objective of the 3Rs is to change traditional animal testing practices, in order to minimize animal testing as much as possible [25]. In addition, applying the 3Rs could minimize animal suffering and distress, increase innovation, and save the costs of traditional animal models [25]. Furthermore, animal research could alter the validity and accuracy of any data attained, because the handling, raising, and treatment of animals can have a strong impact on the physiology and immunology of an animal [26]. Overall, in this case, novel in vitro models would be suitable alternative models to animals for testing toxicity in the future. To better understand the individual toxicity of each toxin, as well as the possible combined outcome upon co-exposure, this review summarizes, based on the available research data, the in vitro toxicity of AFB1 and FB1, and their combined toxicity in different human cells that reflect different target organs.

## 2. Overview of the Toxic Effects of AFB1 In Vitro

Most of the available toxicological knowledge on aflatoxins is related to AFB1. Table 1 summarizes the observed effects of AFB1 in different cell lines for human liver, kidney, intestines, bronchia, male genital system, bone, bone marrow, mammary gland, colon, and brain. Most studies focused on liver, intestine, and kidney as the main toxic effects of AFB1 include hepatotoxicity, enterotoxicity, and nephrotoxicity, respectively. The main selected models to investigate the toxicity of AFB1 in liver and intestine were HepG2 (human hepatocellular carcinoma) cells and Caco-2 (human colorectal adenocarcinoma) cells. HepG2 cells, originally derived from liver biopsies of a 15-year-old Caucasian male with a differentiated hepatocellular carcinoma, are frequently used as an in vitro alternative to primary human hepatocytes for studying the hepatotoxicity of xenobiotics. This is owing to their highly differentiation capability, and displaying many of the genotypic features of normal liver cells [27]. Also, these cells are able to synthesize plasma proteins, bile acid, and glycogen, as well as other functions, such as cholesterol and triglyceride metabolism, lipoprotein metabolism and transport, and insulin signaling. The Caco-2 cells have been applied in various intestinal studies with a high flexibility, high repeatability, and low cost [28]. In particular, as a model of intestinal epithelial barrier, it can spontaneously differentiate into a monolayer of cells with the characteristic of absorbing intestinal epithelial cells, with a brush border layer.

**Figure 3:**
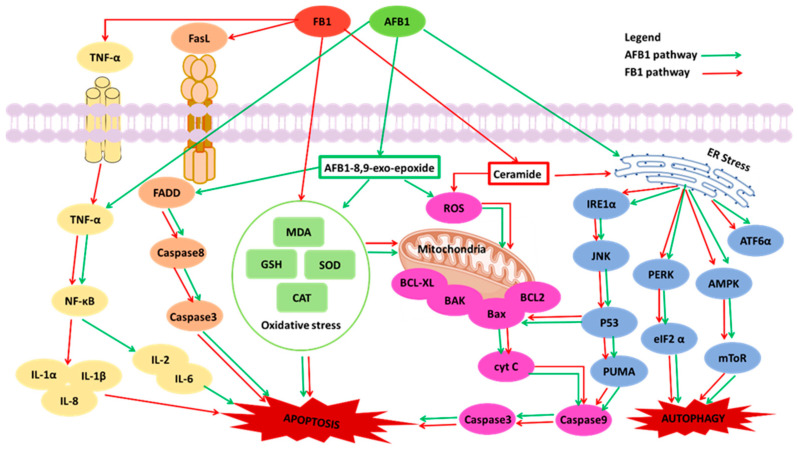
Mechanisms of aflatoxin B1 (AFB1) and fumonisin B1 (FB1) toxicity.

Once it is absorbed by the small intestine, AFB1 is metabolized in hepatic cells by cytochrome CYP450s enzymes, predominantly liver-localized enzymes, to the ultimate carcinogen AFB1-exo-8,9-epoxide [29]. This intermediate highly electrophilic metabolite reacts chemically with DNA and, therefore, causes mutations. However, AFB1 is also metabolized into many hydroxylation compounds through the P450 system, including aflatoxin Q1, aflatoxin P1, aflatoxin B2a, aflatoxin M1, aflatoxicol, and aflatoxicol H1 [29]. Apoptosis or programmed cell death is an evolutionarily conserved mechanism for the selective removal of aging, damaged, or other unwanted cells [30]. This mechanism plays a fundamental role in many physiological processes, and its deregulation can lead to a variety of pathological conditions, including carcinogenesis [30]. In Table 1 and Figure 3, AFB1 mainly activate apoptosis, by inducing several pathways: (1) oxidative stress, (2) mitochondrial pathway, (3) endoplasmic reticulum (ER) stress response, (4) Fas/FasL (Fas ligand) signaling pathway, (5) tumor necrosis factor-alpha (TNF-α) signal pathway (a key cytokine involved in inflammation, immunity, cellular homeostasis, and tumor progression) [31,32,33,34]. Oxidative stress is defined as an imbalance between the increased ROS and a low antioxidant mechanism activity. Increased oxidative stress can lead to damage to the cellular structure [35]. In oxidative stress, AFB1 can decrease antioxidant protein activities (glutathione, superoxide dismutase, and catalase), and increase the concentration of malondialdehyde, to trigger reactive oxygen species (ROS) production [36]. In addition, the oxidative stress caused by AFB1 disrupts mitochondrial function to induce apoptosis, and the manifestation is DNA damage [34,37]. DNA damage can disrupt mitochondrial homeostasis, and induce metabolic pathways resulting in mitochondrial dysfunction [38]. Studies showed that AFB1 increased the expression of anti-apoptotic proteins (Bcl-2 and Bcl-XL), significant mediators of apoptosis (caspase-9, caspase-3, and caspase-8), and decreased the expression of pro-apoptotic proteins (Bax, Bak, and Bid), to induce mitochondrial dysfunction and apoptosis [39]. Recent studies also showed that AFB1 exposure increased the ER stress via the activation of p53, AMP-activated protein kinase, the mammalian target of rapamycin (mTOR), and the c-Jun NH2-terminal kinases [40,41]. Among these activations under the ER stress, AFB1 activated p53 signaling, to disrupt mitochondrial function, to invoke cell apoptosis [39]. High concentrations of AFB1 (100 and 105 μM) suppressed p53 protein expression, and low doses of AFB1 exposure (10 and 16.9 μM) ameliorated this protein expression [42,43,44,45]. From the signaling pathways summarized above, mitochondria were essential mediators of these pathways. In addition to AFB1 impairing organ function by inducing apoptosis through these signaling pathways, the toxin can specifically disrupt cytochrome P450 activities, to trigger liver damage [46,47,48,49]. 

**Table 1:** In vitro toxic effects of aflatoxin B1 (AFB1) in different cell lines after short-term exposure.

Organs	Cells	Exposure Time (Hour)	Concentration (μM)	Effects	References
Liver	HepG2 cells	24	32.0	Inducing cell death, DNA strand breaks, ROS generation, nuclear changes, cell cycle arrests, and apoptotic body formation	[33]
	HepG2 cells	24	13.0	Promoting MDA release, inhibiting cell growth, causing DNA migration, and increasing the level of ERK1/2-P (A) in the MAPK pathway	[41]
	HepG2 cells	24	100.0	Decreasing the expression of the p53 protein	[43]
	HepG2 cells	24	105.0	Suppressing p53 protein expression, and causing mitochondrial damage, nuclear condensation, and a loss of cell-to-cell contact	[45]
	HepG2 cells	24	16.9	Increasing ROS and ΔΨm damage, and the expression of p53	[44]
	HepG2 cells	24	10.0	Inducing ROS production and DNA oxidation	[50]
	HepG2 cells	24	30.0	Increasing GST activity, to induce ROS	[36]
	L-O2 cell	24	192.0	Reducing ΔΨm, and increasing ROS generation	[51]
	HepG2 cells	24	5.0	Causing oxidative stress, and increasing GST activities	[52]
	HepG2 cells	24	30.0	Inducing DNA damage and more significant amounts of ROS	[37]
	HepG2 cells	24	32.0	Inducing oxidative stress, energy metabolism, DNA damage, and cell apoptosis	[34]
	HepG2 cells	24	50.0	Inducing DNA fragmentation and ROS	[53]
	HepG2 cells	24	10.0	Ameliorating DNA damage and p53-mediated apoptosis	[42]
	HepG2 cells	24	10.0	Causing ROS production and DNA damage	[54]
	HepG2 cells	24	10.0	Inducing oxidative lipid damage	[55]
	HL7702 cells	24	10.0	Inducing oxidative stress and DNA damage	[56]
	HepG2 cells	24	10.0	Inducing ROS and DNA strand break, downregulating the Nrf2/HO-1 pathway	[57]
	HepG2 cells	24	4.0	Altering the GSH content, GPx, and SOD activity	[58]
	HepG2 cells	24	3.0	Inducing P450 activities and DNA damage	[46]
	HepG2 cells	24	48.4	Increasing ROS generation and MMP disruption, inducing mitochondrial dysfunction, and inhibiting ATP production	[31]
	L-O2 cell	36	40.0	Inducing autophagy by regulating the EGFR/PI3K-AKT/mTOR signaling pathway	[32]
	HepG2 cells	48	10.0	Decreasing the activity of GST, increasing the P450 3A4 activity, and inducing oxidative stress	[59]
	L-O2 cell	48	8.0	Inducing the expression of P450 and the nuclear translocation of AHR	[48]
	BFH12 cells	48	0.1	Causing lipid peroxidation, reducing the antioxidant activity of the NAD(H): quinone oxidoreductase 1, and increasing the cytochrome P450 3A activity	[47]
	HepG2 cells	72	2.0	Inducing apoptosis and cytochrome P450 1A/1B activity	[60]
Intestine	Caco-2 cells	24	13.0	Promoting MDA release, inhibiting cell growth, causing DNA migration, and increasing the level of ERK1/2-P (A) in the MAPK pathway	[41]
	Caco-2 cells	24	20.0	Leading to cellular apoptosis or necrosis: downregulating the Bcl-2 gene and upregulating the Bax, p53, caspase-3, caspase-8, and caspase- 9 genes, and seriously affecting glycine, serine, threonine, and pyruvate metabolism.	[39]
	Caco-2 cells	24	50.0	Inducing DNA fragmentation and ROS	[53]
	Caco-2 cells	24	10.0	Inducing oxidative lipid damage	[55]
	Caco-2 cells	24	80.6	Increasing ROS and MMP damage, disrupting the ETC, and inhibiting ATP production	[31]
	Caco-2 cells	72	3.0	Increasing intracellular ROS generation, and leading to membrane damage and DNA strand break.	[61]
Kidney	Vero cells	24	40.0	Inducing DNA fragmentation, increasing the level of p53, and decreasing the level of bcl-2 protein	[62]
	HEK cells	24	13.0	Promoting MDA release, inhibiting cell growth, and causing DNA migration	[41]
	PK-15 cells	24	1.0	Inducing ROS production and apoptosis	[63]
	MDCK cells	24	0.8	Inducing oxidative stress: MDA level increased, GSH level and GPX1 activity decreased.	[64]
	HEK 293 cells	48	1.6	Activating oxidative stress	[65]
Bronchial epithelial	BEAS-2B cells	12	1.5	Inducing mutation by the attenuation of DNA adduct and p53-mediated	[66]
	BEAS-2B cells	24	0.1	Inducing apoptosis by inhibiting the CYP enzyme, and increasing DNA adduct	[67]
	BEAS-2B cells	24	1.5	Decreasing both 1A2-expressing and 3A4-expressing CYPs	[68]
Genital system	sperm cells	4	1.0	Decreasing MMP, and inducing fragmented DNA	[69]
Bone marrow	SK-N-SH cells	24	12.8	Promoting MDA release, inhibiting cell growth, and causing DNA migration	[41]
Mammary gland	MAC-T cells	24	12.8	Increasing ROS production, decreasing MMP, and inducing apoptosis, by reducing three anti-stress genes (Nrf2, SOD2, and HSP70) of the Nrf2 pathway	[70]
Bone	MSCs and CD34+ cells	24	10.0	Inducing DNA damage	[71]
Colon	HCT-116 cells	24	10.0	Increasing the expression of p53	[72]
Brain	NHA-SV40LT cells	48	50.0	Inducing cytosolic and mitochondrial calcium changes and ROS generation, and changes in AKT and ERK1/2 MAPK signaling	[40]

ΔΨm: mitochondria membrane permeability; ROS: reactive oxygen species; GST: glutathione S-transferase; DNA: deoxyribonucleic acid; MDA: malondialdehyde; ERK: extracellular signal-regulated protein kinase; MAPK: mitogen-activated protein kinase; GST: glutathione S-transferase; GSH: glutathione; GPx: glutathione peroxidase; CYPs: cytochromes P450; MMP: mitochondrial membrane potential; Nrf: nuclear factor erythroid 2-related factor; HSP: heat shock protein; AKT: protein kinase B; AHR: aryl hydrocarbon receptor; ETC: electron transport chain.

## 3. Overview of the Toxic Effects of FB1 In Vitro

FB1 is a water-soluble molecule, and typically has a low bioavailability (3–6%). It is rapidly distributed in liver and kidney, extensively biotransformed, and rapidly excreted, mostly in feces [73]. It is reported that the hydrolytic biotransformation metabolites, pHFB1 and HFB1, are present in limited amounts in body tissues [73]. FB1 toxicities in cell models of liver, intestine, bone, colon, brain, esophagus, and endothelia are summarized in Table 2. As FB1 toxicities are associated with hepatotoxicity and enterotoxicity, most of these studies (*n* = 14) investigated the effect of FB1 in liver and intestine in which HepG2 and Caco-2 cells were the in vitro models of choice, accounting for 100% and 60%, respectively.

Around 57% of the presented 14 studies indicated that FB1 toxicity was related to the biosynthesis of sphingolipids, which are fundamental components of eukaryotic cells [67]. In addition to playing structural roles in cell membranes (including the synthesis of metabolites of ceramide, sphingosine, and sphingosine-1-phosphate), sphingolipids have attracted attention as bioactive signaling molecules involved in regulating cell growth, differentiation, aging, and apoptosis [74]. As the chemical structure of FB1 resembles sphingolipids, FB1 interferes with the metabolism of sphinganine and sphingosine in the synthesis of ceramide in mitochondria, complicating the sphingolipid biosynthesis pathway, and causing mitochondrial fragmentation [75,76]. Ceramide synthases are integral membrane proteins of the ER, and FB1 could inhibit ceramide synthases [77,78]. Based on the above studies [75,76,77,78], it indicates that FB1 could inhibit ceramide synthases, to affect all pathways and, consequently, invoking cell apoptosis. The mechanisms behind FB1-induced toxicity (Table 2 and Figure 3) include the induction of oxidative stress, the mitochondrial pathway, and ER stress (mTOR) [31,79,80]. In the oxidative stress pathway, FB1 has been shown to induces cytotoxicity, lipid peroxidation, ROS, and DNA damage in cell models of the liver, intestine, brain, and endothelia (Table 2) [81,82,83]. In the mitochondrial pathway, FB1 have the toxic effect to induce mitochondrial dysfunction [31,84]. Chen et al. reported, using Seahorse Respirometry Analysis, that FB1 induced mitochondrial membrane potential (MMP) damage and mitochondrial dysfunction, to disrupt the electron transport chain (ETC), and inhibit ATP production, after exposure for 24 h, in both HepG2 cells and Caco-2 cells [31]. Also, Khan et al. reported an alteration in MMP and ATP production following the exposure of oesophageal (SNO) cancer cells to FB1 for 48 h [84]. In the ER stress pathway, FB1 is attributed to the activation of the IRE1 α -JNK axis, the suppression of mTOR, and the activation of LC3I/II to reduce cellular apoptosis and autophagy in HepG2 cells [80]. In summary, FB1 could inhibit ceramide synthases, induce oxidative stress, disrupt mitochondrial pathway, and suppress the ER stress pathway to show the toxic effects to the human based on the in-vitro data.

**Table 2:** In vitro toxic effects of fumonisin B1 (FB1) in different cell lines after short-term exposure.

Organs	Cells	Exposure Time (Hour)	Concentration (μM)	Effects	References
Liver	HepG2 cells	6	50.0	Reducing ceramide levels, elevating the expression of ABCA1 (a cholesterol efflux promoter) in an LXR-dependent mechanism, and disrupting lipid homeostasis	[85]
	HepG2 cells	24	50.0	Inducing autophagy via the generation of ROS, ER stress, the phosphorylation of JNK, suppressing mTOR, and activating LC3I/II	[80]
	HepG2 cells	24	200.0	Inhibiting sphingolipid biosynthesis and upregulating the anti-apoptotic Birc-8/ILP-2 gene and protein expression to induce apoptosis	[86]
	HepG2 cells	24	35.0	Inducing ROS generation, MMP damage, and mitochondrial dysfunction	[31]
Intestine	HT-29 cells	12	69.0	Inducing lipid peroxidation	[79]
	Caco-2 cells	24	20.0	Inhibiting DNA synthesis	[81]
	Caco-2 cells	24	560.7	Increasing ROS and MMP damage, disrupting the ETC, and inhibiting ATP production	[31]
	Caco-2 cells	48	20.0	Inhibiting sphingolipid biosynthesis	[87]
	LLC-PK1 cells	48	50.0	Inhibiting cell proliferation, and decreasing TEER	[88]
Bone	SH-SY5Y cells	24	50.0	Leading to a sustained deregulation of calcium homeostasis and, presumably, to cell death	[89]
Colon	HT-29 cells	24	50.0	Inhibiting ceramide synthesis and sphingolipids	[90]
Brain	U-118MG cells	48	100.0	Causing ROS production and lipid peroxidation, and lowering GSH levels	[82]
Esophagus	SNO cells	48	20.0	Increasing lipid peroxidation, decreasing GSH, altering mitochondrial membrane depolarization, and depleting ATP	[84]
Endothelia	HUVEC cells	48	50.0	Inducing lipid peroxidation and ROS	[83]

ROS: reactive oxygen species; GST: glutathione S-transferase; ABCA1: ATP binding-cassette A1; LXR: liver X receptors; JNK: c-Jun N-terminal kinase; ILP: inhibitor of apoptosis protein-related-like protein 2; mTOR: the mammalian target of rapamycin; Birc-8: baculoviral IAP repeat containing 8; TEER: transepithelial electrical resistance; DNA: deoxyribonucleic acid; ATP: adenosine triphosphate; LC3: microtubule-associated protein light chain 3; ETC: electron transport chain.

## 4. Combined Toxicity of AFB1 and FB1 in Human Cells

The combined exposure to AFB1 and FB1 is of concern to public health. It has been reported that a synergistic interaction between AFB1 and FB1 is present via the induction of cell apoptosis [91,92]. Du et al. showed a synergistic interaction after HepG2 cell exposure to two sets of combinations: (1) 0.1 μM AFB1 and one μM FB1, (2) 5 μM AFB1 and 85 μM FB1 for 24 h. This synergistic interaction is related to the expression of apoptosis proteins (Bax, Caspase 3, and p53) via immunocytochemistry analysis [91]. Also, the authors reported that the synergetic proapoptotic activity of AFB1 and FB1 was likely caused by different mechanisms, due to the expression of the antagonistic caspase 8 [91]. In addition, the study by Mary et al., suggested a possible synergistic interaction toward genotoxicity in BRL-3A cells a mixture of AFB1 (20 μM) and FB1 (30 μM) after 48 h. including an increase in the arachidonic acid metabolism, cytochrome P450 activity, and p53 protein levels [92]. In this interaction, they argued that AFB1 had a major input into the mixture’s prooxidant activity, with cytochrome P450 and arachidonic acid being ROS contributors, but that FB1 was weak at invoking these pathways [92]. Chen et al. have also reported that the mixture of AFB1 (25.6 μM) and FB1 (224 μM) significantly increased the p53 protein, and downregulated the mitochondrial complexes in HepG2 cells [93]. Although the selected concentrations in the binary mixture of AFB1 and FB1 is different than the above mentioned studies, the ratio of both toxins is less than 20, and the synergistic interaction is still valid in hepatocytes. In addition, the same authors demonstrated that FB1 is contributing more than AFB1 to the mixture effects, based on RNA transcriptomic analysis [93], which is consistent with previous studies that showed that the binary mixture of AFB1 and FB1 would synergistically raise the hepatocarcinogenic properties. As shown in Figure 3, with AFB1 and FB1 having different mechanisms of action, there could be a potential of promoting each other via crossing pathways. In liver tumors, when AFB1 and FB1 were combined, the disruption of sphingolipid metabolism was promoted, which suggested that alterations in the associated sphingolipid signaling pathways were potentially responsible for the promotional activity of FB1 toward AFB1 [94]. Furthermore, FB1 could promote hepatocarcinogenesis when co-exposed to along with AFB1 [94]. Similarly, Torres et al. stated that FB1 has a potential to modulate AFB1 hepatoxicity, because FB1 could inhibit ceramide synthases, and the inhibition of sphingolipid signaling pathways could contribute to the tumorigenicity of AFB1 [95]. Therefore, within some ranges of combined AFB1 and FB1, they could cause synergistic toxicity in humans. At a lower ratio of combination (lower than 20) for both mycotoxins, the interaction is synergistic in the process of apoptosis in hepatic cells, such as the expression of the apoptosis-associate Bax and Bcl-2 proteins. However, when the combined ratio is slightly higher, the interaction of the two mycotoxins would no longer show an apparent synergistic effect but gradually tend toward an additive effect [91]. The combination of AFB1 (10 μM) and FB1 (300 μM) only increased the Bax, Caspase-8, Caspase-3, and p53, without a synergistic effect in HepG2 cells, and the combined ratio of AFB1 and FB1 is 30 (FB1/AFB1). On the other hand, an antagonistic interaction between AFB1 and FB1 may happen. McKean et al. mentioned a weak antagonistic effect in HepG2 cells of AFB1 and FB1 [96]. The combined AFB1 (1 μM) and FB1 (399 μM) did not reduce the cell viability of HepG2 cells after 24 h, and this combination ratio (FB1: 399 μM/AFB1: 1 μM = 399) is the highest applied in vitro concentrations found in the literature [96]. The summarized data showed that the combined ratio of AFB1 and FB1 could be the main parameter that affects the interaction of both toxins in hepatic cells. In their study, a strong additive interaction was found in BEAS-2B (human bronchial epithelial) cells after exposure to the combined AFB1 (100 μM) and FB1 (355.1 μM) over 24 h [96]. The interaction between these two toxins would vary, depending on the organs. These findings indicate that the interaction of AFB1 and FB1 is mainly manifested as a synergistic effect, and the additive/synergistic effect is primarily regulated by their ratio and organs. Therefore, the AFB1 and FB1 mixture may enhance toxic effects, and carry a more significant risk factor than their individual presence.

## 5. Mycotoxin Mitigation

**Figure 4:**
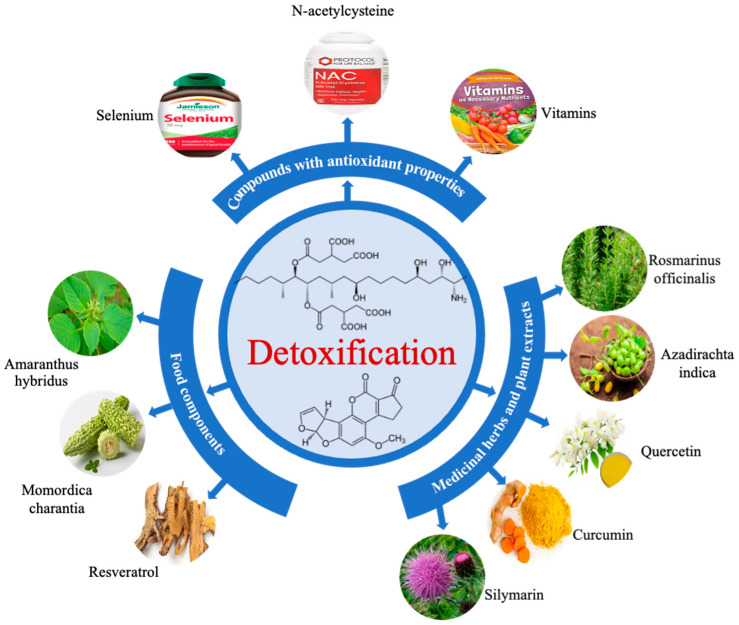
Mitigation strategies against AFB1 and FB1 toxicity.

As human exposure to AFB1 and FB1 results in several serious toxicological effects, mitigating both mycotoxins is a prerequisite. Several compounds with antioxidant properties, food components, and medicinal herbs and plant extracts have been proposed based on their potential efficacious effects to alleviate AFB1 and/or FB1 toxicity in vitro. As shown in Figure 4, compounds with antioxidant properties that reduce AFB1 and/or FB1 toxicity contain selenium, N-acetylcysteine, and vitamins [97,98,99,100,101,102,103,104]. Selenium may ameliorate AFB1-induced hepatic dysfunction or damage and modulated the expression of apoptotic related proteins (Bcl-2, Bax, caspase-3, and p53) after three weeks of treatment [98,99]. Unlike selenium, N-acetylcysteine mitigated AFB1 toxicity by increasing the formation of glutamyl glucoside peptides in porcine kidney-15 cells and reduced the oxidative damage, inhibited the apoptosis, and regulated the mRNA expression of Bax, Bcl-2, caspase-3, caspase-9, cytochrome c and P53 induced by FB1 in the liver and kidney [100,101]. Vitamins, including A, C, and E, could also reduce the oxidative damage induced by AFB1 in human lymphocytes, especially inhibiting AFB1-induced ROS generation [102,103]. In addition, vitamin A and vitamin C could inhibit the formation of AFB1-DNA adducts, and vitamin E enhanced covalent binding of AFB1 to DNA in hepatocytes [104]. On other hand, vitamin E was reported to prevent DNA fragmentation and apoptosis induced by FB1 in human glioma cells [105].

In fact, some food components not only keep the body’s systems functioning properly, but also mitigate the toxic effects of mycotoxins including AFB1 and FB1. Among these components, *Amaranthus hybridus*, Resveratrol, and Momordica charantia have been reported the mitigation capability of both toxicity in different ways (Figure 4). *Amaranthus hybridus* (traditional African vegetable) extract was reported a protective effect against AFB1 and FB1 that induced cytotoxicity and DNA damage and induced genotoxicity in hepatoma cells [106,107]. Resveratrol, mainly derived from peanuts, and grapes, could also alleviate AFB1-induced cytotoxicity, including the increase in ROS, the decrease in MMP and apoptosis and exhibiting a good regulatory effect on components of the Nrf2 signaling pathway (including Nrf2, Keap1, NQO1, HO-1, SOD2 and HSP70) in bovine mammary epithelial cells [68]. Besides, Momordica charantia, a popular vegetable, has been claimed to contain many potent mitigation compounds to induce the toxicity of AFB1, but its exact composition of these compounds are still unknown [108].

Medicinal herbs and plants also contain many natural components that were used in the prevention, treatment, diagnosis, rehabilitation and health care of diseases, including the capability to counteract the AFB1 and FB1 toxicity [109]. Natural compounds that are extracted from Rosmarinus officinalis and Azadirachta indica var. siamensis could inhibit DNA adduct formation and reduce metabolic activation of AFB1 to mitigate AFB1 toxicity in hepatoma cells [108,110]. Besides, quercetin could reduce AFB1-induced lipid peroxidation and reverted cytochromes P450 variations to show its mitigation capability in the liver [111]. Recently, Elbasuni et al. also proved that Chlorella vulgaris could mitigate hepatic aflatoxicosis [111]. They found that Chlorella vulgaris mitigated AFB1-induced oxidative stress and inflammatory condition after three week treatment [112] On other hand, curcumin and silymarin have been studied to have the capability to provide cytoprotection against toxicity induced by FB1 and specially to reduce ROS formation after 48h treatment in porcine kidney-15 cells [113]. From the above, compounds with antioxidant properties, food components, and medicinal herbs and plant extracts are universal choices to mitigate the hepatic and nephric AFB1- and FB1- toxicosis in human beings mainly by reducing oxidative damage, DNA fragmentation, and apoptosis.

## 6. Conclusions

In the last few decades, several efforts have been made to minimize exposure to mycotoxins, especially AFB1 and FB1, from food. Despite all the attempts to control their content in food, the research has not fully succeeded in solving this major problem. In addition, several underlying toxic mechanisms have not been completely unraveled. Therefore, there is a need to deeply investigate their toxicities by implementing state-of-the art methodology, such as Omic technologies. The review shows that AFB1 and FB1 could invoke, after short-term exposure, cell apoptosis, by inducing several pathways (oxidative stress, the mitochondrial pathway, ER stress, the Fas/FasL signaling pathway, and the TNF-α signal pathway) in different cell models (mainly in HepG2 cells and Caco-2 cells). The combination of AFB1 and FB1 is mainly manifested as a synergistic effect, and their interaction is mainly related to the FB1/AFB1 ratio and the organs. However, the in vitro toxicity work was performed using two-dimensional (2D) models, or the cell monolayer. More advanced three-dimensional (3D) in vitro models, such as organoids and spheroids, which exhibit features that are closer to the complex in vivo conditions, have not been adequately used in mycotoxin field. The 3D culture models have proven to be more realistic for translating the study findings for in vivo applications. To better understand the toxicity of AFB1 and FB1 in vitro, the use of 3D models should be increased, to study various aspects of cell physiology and pathology in the future. Additionally, investigations on the effects of long-term exposure to low doses of AFB1 and FB1 should receive more attention, as humans are more likely to be exposed to low doses on a daily basis from food. Finally, due to insufficient data, the mechanisms of interaction still need to be elucidated. In the future, more combined ratios of AFB1 and FB1, and more pathways proposed and target proteins for their combined toxicity, should be observed, to support this synergistic interaction between AFB1 and FB1. Moreover, focusing on the numerous organ models for their combined toxicity would fill the knowledge gaps around the currently uncertain hazards for human health.

## Data Availability

Not available.
